# Beclin 1 and UVRAG Confer Protection from Radiation-Induced DNA Damage and Maintain Centrosome Stability in Colorectal Cancer Cells

**DOI:** 10.1371/journal.pone.0100819

**Published:** 2014-06-23

**Authors:** Jae Myung Park, David Tougeron, Shengbing Huang, Koichi Okamoto, Frank A. Sinicrope

**Affiliations:** Mayo Clinic and Mayo Clinic Cancer Center, Rochester, Minnesota, United States of America; Swedish Medical Center, United States of America

## Abstract

Beclin 1 interacts with UV-irradiation-resistance-associated gene (UVRAG) to form core complexes that induce autophagy. While cells with defective autophagy are prone to genomic instability that contributes to tumorigenesis, it is unknown whether Beclin1 or UVRAG can regulate the DNA damage/repair response to cancer treatment in established tumor cells. We found that siRNA knockdown of *Beclin 1* or *UVRAG* can increase radiation-induced DNA double strand breaks (DSBs), shown by pATM and γH2Ax, and promote colorectal cancer cell death. Furthermore, knockdown of *Beclin 1*, *UVRAG* or *ATG5* increased the percentage of irradiated cells with nuclear foci expressing 53BP1, a marker of nonhomologous end joining but not RAD51 (homologous recombination), compared to control siRNA. *Beclin 1* siRNA was shown to attenuate UVRAG expression. Cells with a *UVRAG* deletion mutant defective in Beclin 1 binding showed increased radiation-induced DSBs and cell death compared to cells with ectopic wild-type *UVRAG*. Knockdown of *Beclin 1* or *UVRAG,* but not *ATG5,* resulted in a significant increase in centrosome number (γ-tubulin staining) in irradiated cells compared to control siRNA. Taken together, these data indicate that Beclin 1 and UVRAG confer protection against radiation-induced DNA DSBs and may maintain centrosome stability in established tumor cells.

## Introduction

Macroautophagy is a catabolic, lysosomal degradation pathway that maintains cellular biosynthesis during metabolic, hypoxic, or cytotoxic stress [1]. A key regulator of autophagy is Beclin 1 whose protein is a core component of the class III PI3K/Vps34 complex that is required for autophagosome formation and maturation [2]. Beclin 1 interacts with several proteins including autophagy regulators, organelle membrane anchor proteins, and Bcl-2 and Bcl-x_L_. A coiled-coil domain in Beclin 1 serves as a protein interaction platform to recruit two major autophagy regulators, Atg14 and UV radiation resistance-associated gene (*UVRAG*) product [3]. UVRAG, originally identified through its ability to complement UV-radiation sensitivity in tumor cells, associates with the Beclin 1-Bcl-2-PI(3)KC3 multiprotein complex where it and Beclin 1 interact via their coil coil domain (CCD) and interdependently induce autophagy [4]. *Beclin 1* and *UVRAG* function as tumor suppressor genes, and *Beclin 1^+/−^* mice were shown to be tumor-prone [5]. Beclin 1 maps to a region on chromosome 17q21, and *Beclin 1* [6] and *UVRAG* [7] are monoallelically deleted in certain cancers. Allelic loss of *Beclin 1* and defective autophagy were shown to sensitize cells to metabolic stress [8], and to activate the DNA damage response in association with aneuploidy in immortalized murine epithelial cells and in mammary tumors [8].

In established tumors, basal autophagy is upregulated to survive metabolic, hypoxic or cytotoxic therapy-related stress, indicating that autophagy can serve as a mechanism of therapeutic resistance [9]. Autophagy inhibition has been shown to increase cancer cell sensitivity to chemotherapy or radiation, establishing autophagy as a novel target for therapy [10], [11]. Recent data indicate that cells with defective autophagy are prone to genomic instability with increased DNA damage and aneuploidy [8], [12]. However, evidence supporting a role for autophagy in genome protection in established cancers is limited and the role of Beclin 1, if any, is unknown. It has been reported that UVRAG plays a dual role in chromosomal stability that was found to be independent of autophagy [13]. Cancer therapies induce DNA double-strand breaks (DSBs) that activate DNA repair mechanisms including non-homologous end joining (NHEJ) and homologous recombination (HR) to restore genomic integrity [14]. Recent data indicate that UVRAG can promote DNA DSB repair by directly binding and activating DNA-PK in NHEJ [13]. Histone H2Ax, a substrate of ataxia telangiectasia mutated (ATM) and DNA-dependent protein kinase (DNA-PK) (key enzyme in NHEJ), is phosphorylated on serine 139 and forms foci on DSB sites that can serve as a marker of DSBs [15]. Maintenance of genomic integrity requires proper chromosome segregation during cell division that is largely dependent upon assembly of the mitotic spindle apparatus by centrosomes. Extra centrosomes almost inevitably cause spindle malformation and erroneous chromosomal segregation [16] that in response to DNA damage, can lead to aneuploidy and genomic instability [17]. Defects in genes involved in DNA repair have been shown to cause aberrations in centrosome number that is common in human tumors [18].

Although the role of Beclin 1 and UVRAG have been studied in the setting of tumorigenesis [4], [13], [19], little is known about their role in the regulation of genomic stability and the potential importance of their interaction in this process in established tumors. To gain insight into the mechanism(s) by which tumor cell autophagy can confer treatment resistance, we examined the ability of Beclin 1 and/or its cofactor UVRAG to regulate the DNA damage response and centrosome number in colorectal cancer (CRC) cell lines. CRCs are highly resistant to DNA damaging therapies such as cytotoxic chemotherapy and radiation which are commonly given concurrently in the clinic. In this regard, we previously reported that Beclin 1 overexpression was associated with reduced survival in colon cancer patients treated with 5-fluorouracil as adjuvant therapy [20]. In the current study, we found that Beclin 1 and UVRAG interact to regulate DNA damage/repair that utilizes non homologous end joining and maintains centrosome stability in response to radiation. A *UVRAG* deletion mutant defective in Beclin 1 binding failed to protect against DSBs demonstrating the importance of their interaction in the maintenance of genomic stability.

## Materials and Methods

### Cell Culture, Drugs, Reagents, and Cell Radiation

Human colorectal cancer cell lines, HT-29 and DLD1, and HeLa cervical carcinoma cells were cultured in RPMI 1640 (Invitrogen) supplemented with 10% fetal bovine serum and 1% antibiotic/antimycotic. 293T cells were grown in DMEM (Sigma Chemical Co.) and supplemented as above. All cell lines were purchased from American Type Culture Collection (ATCC) and described previously [21], [22], with exception of Hela cells that were obtained from Dr. S. Kaufmann at Mayo Clinic [23]. Cells were treated with 5-fluorouracil, bafilomycin A1 (Sigma, B1793), and spautin-1 (Cellagen Technology, C3430-2s) as indicated. Drugs were dissolved in dimethyl sulfoxide (DMSO) which was also used as a treatment control. Cells were also treated with ionizing radiation with a Cesium 137 source on a MARK 1–25 irradiator (JL Shepherd and Associates).

### Small Interfering RNA (siRNA)

Cells were transfected with siRNA oligonucleotides using Lipofectamine RNAiMAX Reagent (Invitrogen) and targeting sequences for *Beclin 1* (GGGTCTAAGACGTCCAACA), *cytosolic-associated protein light chain 3 B* (*LC3B*) (GAAGGCGCTTACAGCTCAA), *UVRAG* (TCACTTGTGTAGTACTGAA), and *autophagy protein 5* (*ATG5*) (GGCATTATCCAATTGGTTT) according to manufacturers’ protocol. To perform double knockdown, cells were transfected with two siRNA oligonucleotides targeting different proteins, 24 h in between for cells to recover.

### Lentiviral Short Hairpin RNA

Short hairpin RNA (shRNA) template oligonucleotides (synthesized by the Mayo Clinic Molecular Biology Core Facility) were ligated into the lentiviral shRNA cloning and expression vector pSIH1-H1 (System Bioscience, Mountain View, CA. The control shRNA sequence was CAACAAGATGAAGAGCACCAA (Sigma). The targeting sequence for ubiquitin-specific peptidase 10 (*USP10)* was GCCTCTCTTTAGTGGCTCTTT. Lentivirus production using 293T cells and transduction of target cells were performed as previously described [24]. Puromycin (2 µg/mL; Sigma, P8833) was added at 48 h post-transduction, and puromycin-resistant cells were utilized.

### Ectopic Expression of Retroviral *UVRAG*

cDNA of *UVRAG* (Origene) was subcloned into pBabe-puro vector with an N-terminal 3-tag. The generation of a mutated *UVRAG* with deletion of the coil-coil domain (*ΔCCD*) [25] was achieved by PCR utilizing overlapping primers that spanned the region of interest. Pseudo-typed retrovirus was produced using these *UVRAG* constructs per a previously described procedure [24]. Amino-acids 144–269 were deleted to obtain the *UVRAG ΔCCD* mutant.

### Cell Viability and Apoptosis Assays

The 3–4,5-dimethylthiazol-2-yl)-5-(3-carboxymethoxyphenly)-2-(4-sulfophenyl)-2H-tetrazolium (MTS) colorimetric assay was used to measure cell viability. Apoptosis assay was performed using annexin V/PI staining and caspase-3 cleavage, as previously described [22].

### Immunoblotting

Protein samples were prepared and then loaded onto an SDS-PAGE gel with electrophoretic transfer onto a polyvinylidene difluoride (PVDF) membrane (Bio-Rad), as previously described [24]. Antibodies against the following proteins were utilized: Beclin 1, cleaved caspase-3, γH2Ax, H2Ax, pCHK2, LC3, UVRAG, ATM (all from Cell Signaling Technology, 1∶1000), γ-tubulin, pATM (EPITOMICS, 1∶2000) and p62 (MBL, 1∶2000). Protein bands are quantified and normalized against γ-tubulin using ImageJ (National Institute of Health).

### Clonogenic Survival Assay

Two hundred cells were seeded into each well of a six-well plate and then irradiated (4 Gy) alone or in the presence of 5-fluorouracil (5-FU) (2 µM). After incubation for 7–14 days, cells were fixed with 10% methanol/10% acetic acid and then stained with 0.4% crystal violet in 10% methanol. The number of colonies with >50 cells was determined and expressed as the relative change in drug-treated *vs* untreated cells.

### Immunoprecipitation

Cells were lysed in CHAPS buffer [5 mmol/L MgCl_2_, 137 mmol/L KCl, 1 mmol/L EDTA, 1 mmol/L EGTA, 1% CHAPS, 10 mmol/L HEPES (pH 7.5)] for 30 min on ice and then clarified by centrifugation at 17,000 g for 10 min at 4°C. Lysates were incubated with an antibody overnight at 4°C. Antigen/antibody complex were captured using magnetic protein A/G beads (Thermo Scientific) for 2 h at 4°C. Unbound proteins were washed three times with 1 mL CHAPS buffer without protease inhibitors. Bound proteins on beads were eluted by incubating in LDS sample buffer for 10 min and were subsequently loaded for immunoblotting.

### Immunofluorescence and Confocal Microscopy

Cells were cultured in glass-bottom dishes coated with poly-L-lysine (MatTeck Corp.) and subsequently exposed to γ-radiation (2–8 Gy). Cells were fixed for 10 min with 4% paraformaldehyde, permeabilized with 0.5% Triton X-100 in PBS, and blocked in 3% bovine serum albumin (BSA). Next, cells were stained with primary antibodies against 53BP1 (Cell Signaling Technology, 4937, 1∶100), or RAD51 (Calbiochem, PC130, 1∶100), followed by corresponding fluorescent secondary antibodies (Alexa Fluor 488 or 568, Molecular Probes, Invitrogen). Samples were rinsed, immersed in 0.05 µg/mL 4′,6-diamidino-2-phenylindole (DAPI) for 5 min, and mounted with coverslips using Prolong Gold (Invitrogen). Fluorescence confocal microscopy was performed using an Axiovert 100 M microscope equipped with a Plan-Apochromat 63 X/1.4 objective lens and Zeiss LSM510 software (Carl Zeiss). The percentage of cells containing more than 10 nuclear fluorescent foci per total cell number was calculated by examining a minimum of 100 cells in five fields at 63X for each experimental condition.

For staining of centrosomes, cytoplasmic tubulin was depleted with a microtubule stabilization buffer (3 moll/L EGTA, 50 mmol/L Pipes, 1 mmol/L MgSO_4_, 25 mmol/L KCl). Cells were fixed for 10 min in −20°C methanol [26], permeabilized with 0.1% Triton X-100 in PBS, and incubated in a blocking buffer (5% goat serum, 1% glycerol, 0.1% BSA, 0.1% fish skin gelatin). Next, cells were stained with a primary antibody against γ-tubulin (Sigma, GTU-88, 1∶5000) followed by corresponding fluorescent secondary antibodies. The percentage of cells with more than 2 centrosomes was determined using a fluorescent microscope whereby at least 100 cells in five fields at 63X were counted for each experimental condition.

### Statistical Analysis

Statistical comparisons for experiments in cultured cells were performed using the Student’s t-test. Statistical tests were two-sided with a significance level defined at *P<*0.05.

## Results

### Cytoprotective Effect of *Beclin 1* in Cells Exposed to 5-FU and/or Radiation

**Figure 1:**
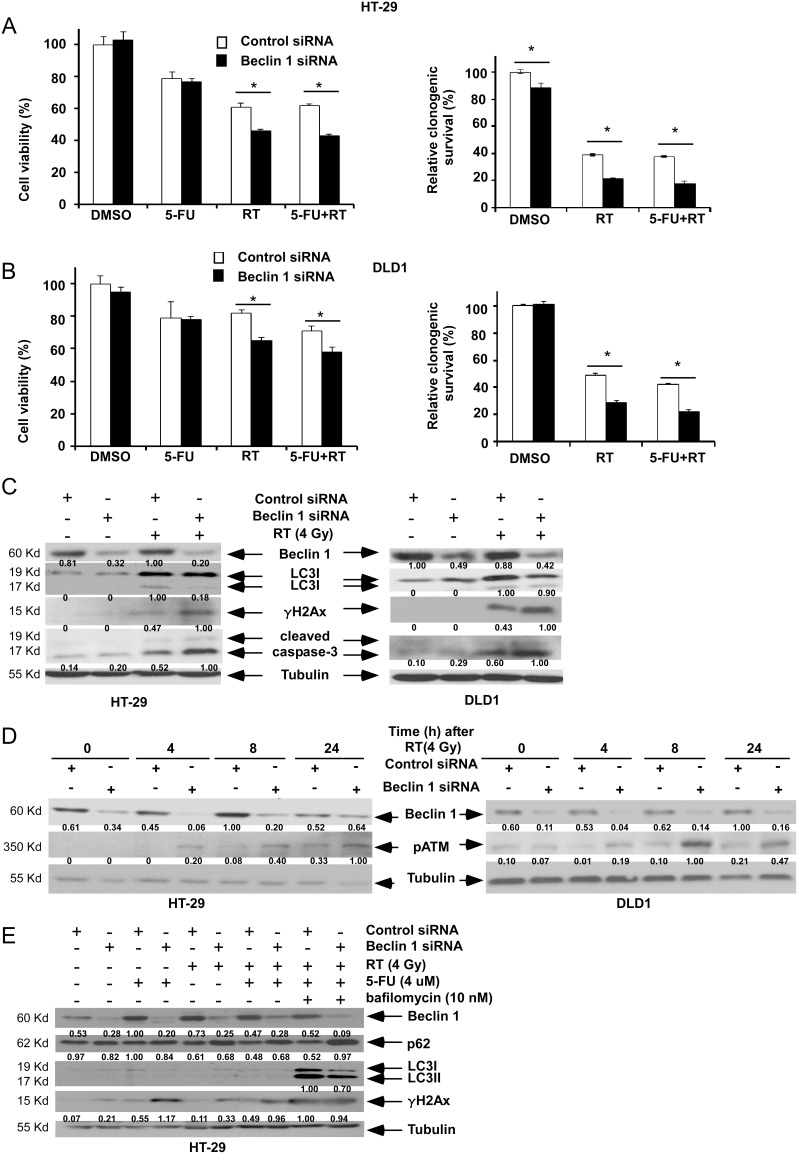
*Beclin 1* suppression enhances radiation-induced DNA damage and cell death. *A, B,* HT-29 (A) or DLD1 (B) cells were transfected with *Beclin 1* vs control siRNA and treated with radiation (RT; 4 Gy) alone or combined with 5-FU (2 µM). Results of the MTS (24 h) and clonogenic survival assays are shown. Data are presented as mean ± standard deviation for experiments performed in triplicate. Statistical significance was determined by a two-sided Student’s t test and defined as *P<0.05. *C*, Cells with *Beclin 1* or control siRNA were irradiated (4 Gy) and then were probed for expression of LC3I-II, γH2Ax, and cleaved caspase-3 by immunoblotting at 24 h. Protein bands are quantified and relative intensity was labelled underneath the corresponding blot. Only LC3II was quantified for LC3 protein. *D,* Time course of the effect of radiation on pATM expression in cells with *Beclin 1* siRNA vs control siRNA. *E*, Effect of *Beclin 1* siRNA on autophagic flux in cells that were treated with RT (4 Gy) and/or 5-FU (4 µM).

We determined whether suppression of Beclin 1 can enhance chemoradiation-induced cytotoxicity. Colon cancer cell lines were treated with γ-radiation (4 Gy) alone or combined with 5-FU (2 µM). In treated cells, *Beclin 1* knockdown *vs* control siRNA was shown to significantly reduce cell viability (Fig. 1A, B, *left panels*). *Beclin 1* knockdown also decreased long-term clonogenic cell survival after radiation ± 5-FU compared to cells with control siRNA (Fig. 1A, B, *right panels*). In these experiments, addition of a clinically achievable dose of 5-FU had a minimal effect on the extent of radiation-induced cell death.

To determine whether Beclin 1 can regulate the DNA damage response, we examined the effect of radiation upon expression of the DSB markers phosphorylated histone H2Ax (γH2Ax) [27] and phosphorylated ataxia telangiectasia mutated (pATM). ATM is a critical sensor of DNA damage that is involved in DNA repair and G2-to-M checkpoint control [28], and whose activation requires its autophosphorylation [29]. Suppression of *Beclin 1* by siRNA increased γH2Ax expression two-fold, induced pATM, and increased caspase-3 cleavage (2-fold) in cells exposed to radiation compared to control siRNA (Fig. 1C, D). Modulation of γH2AX at 24 hr likely represents non-repaired, residual DNA damage post irradiation. We then determined whether *Beclin 1* siRNA can inhibit radiation-induced autophagic flux. Using bafilomycin A1 that inhibits vacuolar H+ ATPase, we observed the accumulation of cytosolic (LC3I) and membrane bound (LC3II) forms of LC3 (Fig. 1E) whose ratio is correlated with the extent of autophagosome formation [1], [30]. In cells treated with 5-FU + radiation and bafilomycin A1, *Beclin 1* knockdown was shown to attenuate the accumulation of LC3I-II compared to control cells and to increase expression of the autophagy substrate p62/sequestosome1 [24] consistent with inhibition of autophagic flux (Fig. 1E).

**Figure 2:**
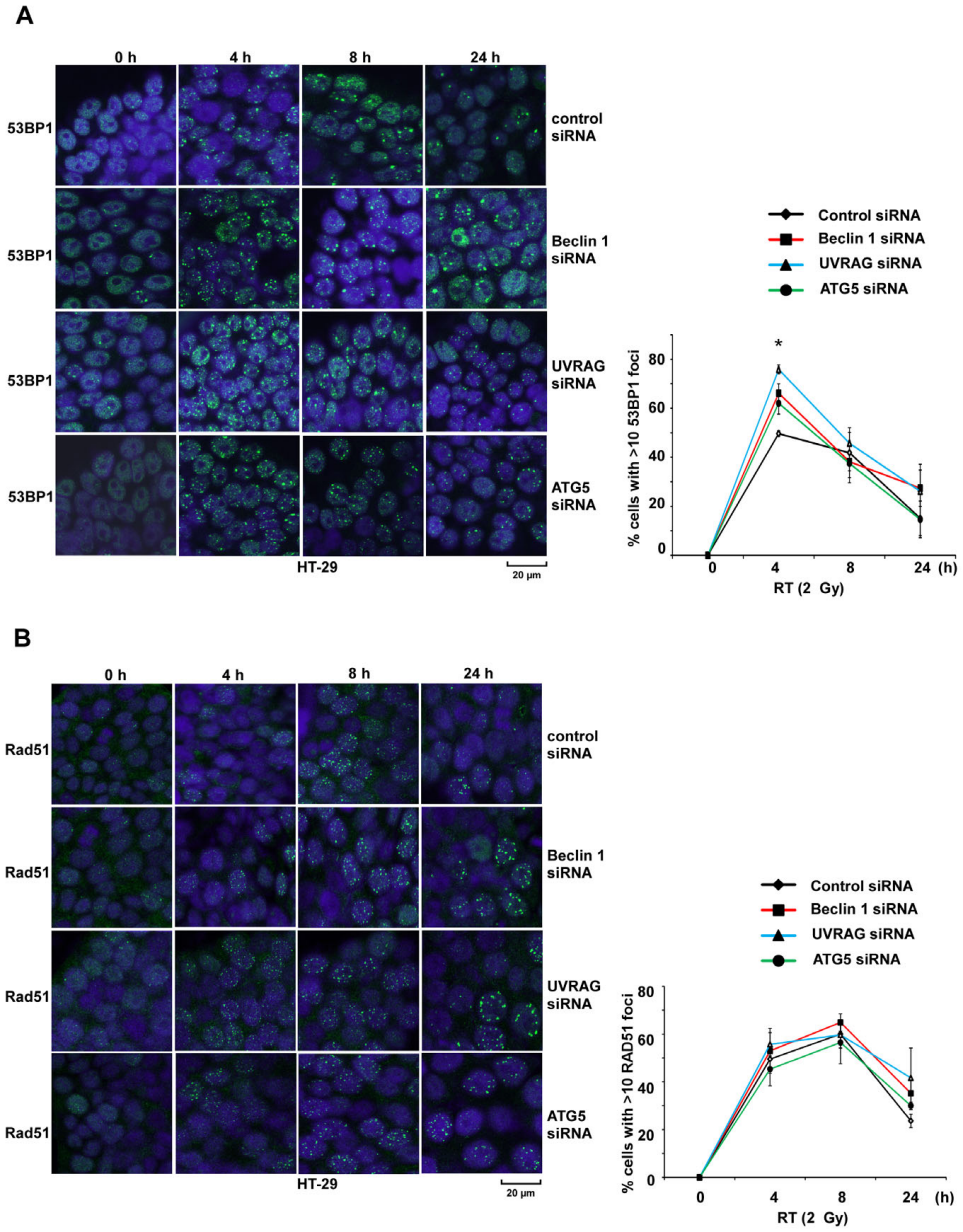
Knockdown of *Beclin 1, UVRAG, and ATG5* increase radiation-induced 53BP1, but not RAD51, nuclear foci. *A, B,* Immunofluorescence staining for 53BP1 (A) or RAD51 (B) was performed in HT-29 cells with knockdown of *Beclin 1*, *UVRAG* or *ATG5* and exposed to radiation (2 Gy) at the indicated times. The percentage of cells with >10 nuclear foci expressing either 53BP1 or RAD51 was calculated and plotted as shown. 53BP1 and RAD51 are markers of nonhomologous end joining and homologous recombination, respectively. DAPI was utilized to counterstain the nucleus. Data are presented as mean ± standard deviation for experiments performed in triplicate. Statistical significance was determined by a two-sided Student’s t test and defined as *P<0.05.

We then determined the ability of Beclin 1 and UVRAG to modulate the DNA damage response in irradiated cells. Expression of 53BP1 is a sensor for DNA damage and a facilitator of NHEJ [31] whereas RAD51 is a critical regulator of DNA repair through HR [32]. Irradiation was associated with an increase in the percentage of cells with >10 53BP1 nuclear foci after 4 hours that was significantly (p<0.05) enhanced in *Beclin 1*, *UVRAG* or *ATG5* knockdown *vs* control cells (Fig. 2A). *ATG5* knockdown cells were utilized as a control for disabling autophagy. Irradiation also increased the number of RAD51 foci which did not differ significantly among *Beclin 1*, *UVRAG* or *ATG5* knockdown *vs* control cells (Fig. 2B).

**Figure 3:**
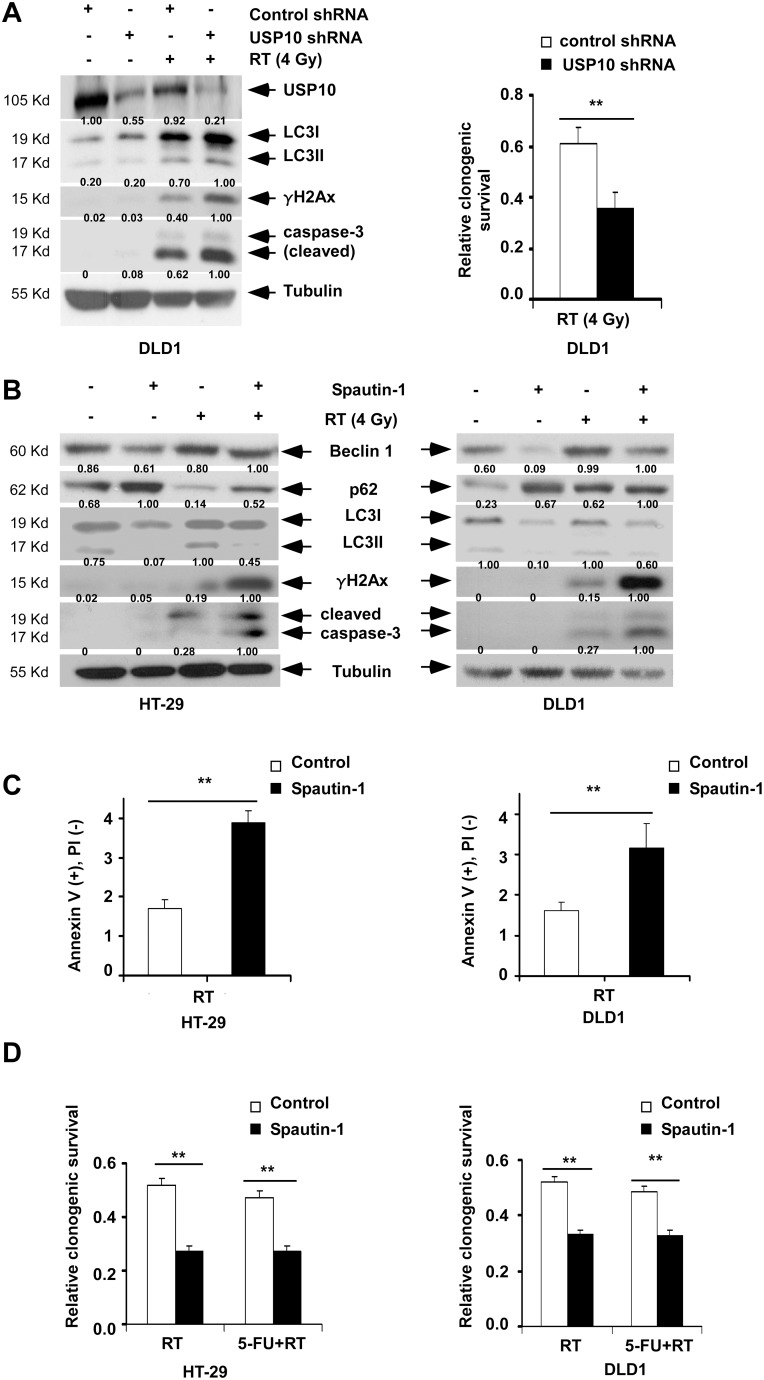
Autophagy inhibition by *USP10* shRNA or spautin-1 enhances irradiation-induced DNA damage and cell death. *A,* Cells with *USP10 vs* control shRNA were irradiated and analyzed for expression of USP10, LC3I-II, γH2Ax and cleaved caspase-3 at 24 h by immunoblotting (*left*), or for clonogenic survival (*right*). *B,* Cells were treated with irradiation alone or combined with spautin-1 (10 µM) and expression of the indicated proteins were analyzed at 24 h by immunoblotting. *C, D,* Cells were treated with spautin-1 (10 µM) alone or combined with RT (4 Gy) (C) or RT ± 5-FU (2 µM) (D). In these cells, annexin V^+^PI^−^ labeling at 24 h (C) and clonogenic survival (D) were analyzed. Data are presented as mean ± standard deviation compared to controls for triplicate experiments. **P<0.01.

USP10 and USP13 have been shown to mediate the deubiquitination of Beclin 1, thereby stabilizing the Vps34 complex [33]. Inhibition of these deubiquitinases may, therefore, represent a strategy to suppress autophagy. We found that suppression of *USP10* by shRNA induced a 2-fold increase in the DSB marker γH2Ax and caspase-3 cleavage, and also reduced clonogenic survival in irradiated cells (Fig. 3A). Inhibition of USP10 and USP13 was also accomplished using spautin-1, a potent small molecule inhibitor of autophagy that promotes degradation of Vps34 PI3 kinase complexes [33]. Spautin-1 inhibited autophagy as indicated by reduced LC3I-II conversion and accumulation of p62/sequestosome 1 (Fig. 3B). Furthermore, spautin-1 enhanced DSBs, modestly induced apoptosis (Fig. 3B, C), and reduced clonogenic survival in cells exposed to radiation alone or combined with 5-FU (Fig. 3D).

### *UVRAG* Interacts with *Beclin 1* to Regulate the DNA Damage Response

**Figure 4:**
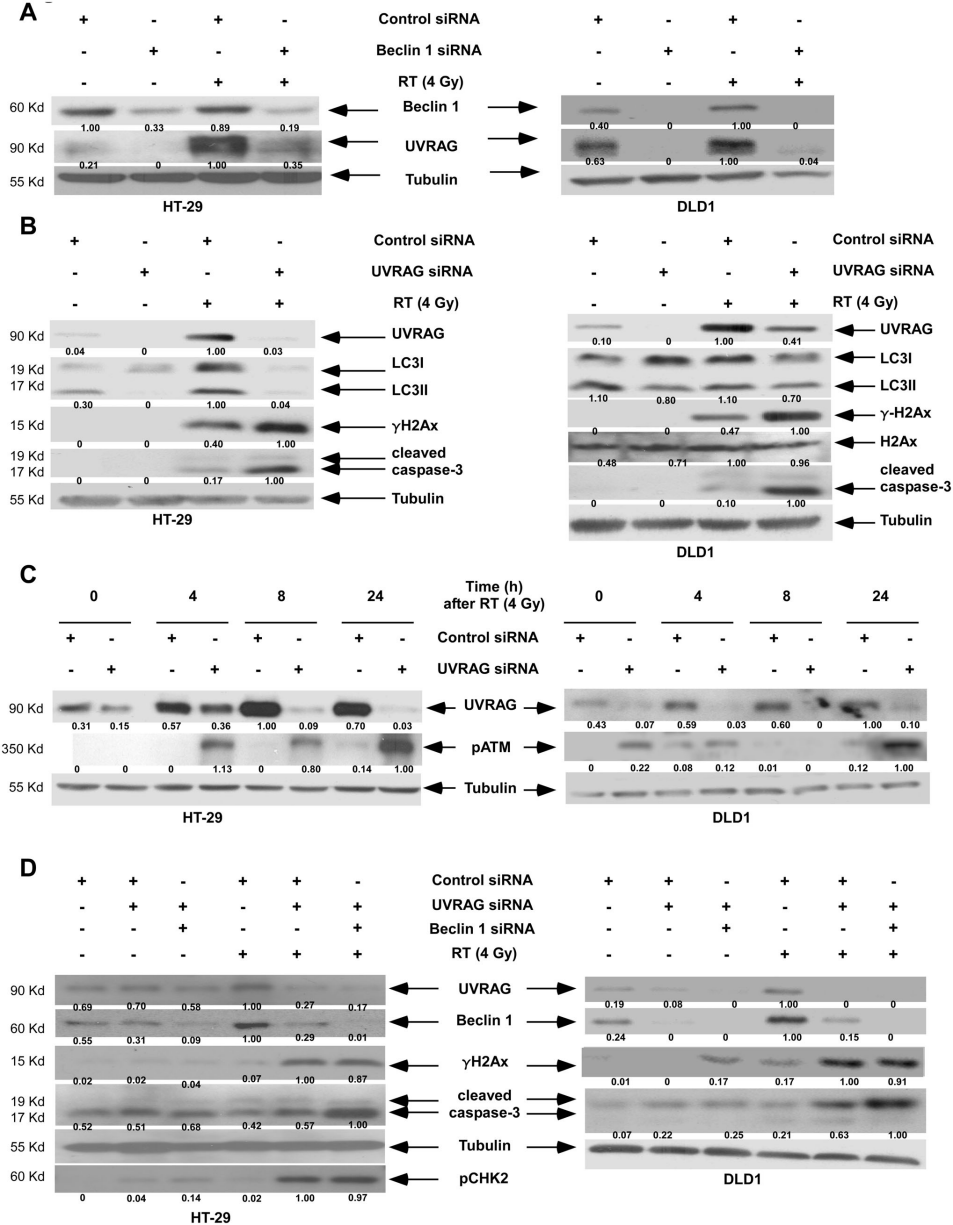
Suppression of *Beclin 1, UVRAG* or both sensitize cells to DNA damage and apoptosis. *A, B,* HT-29 and DLD1 cells were transfected with *Beclin 1* (A) or *UVRAG* (B) *vs* control siRNA. Cells were irradiated and the expression of the indicated proteins was analyzed at 24 h post-radiation by immunoblotting. *C,* Time course of the effect of radiation on pATM expression in cells with *UVRAG vs* control siRNA. *D,* Effect of radiation on DNA damage and apoptosis markers in cells with dual knockdown of *Beclin 1* and *UVRAG* vs *UVRAG* siRNA alone (24 h). Densitometry was performed and normalized against tubulin.

**Figure 5:**
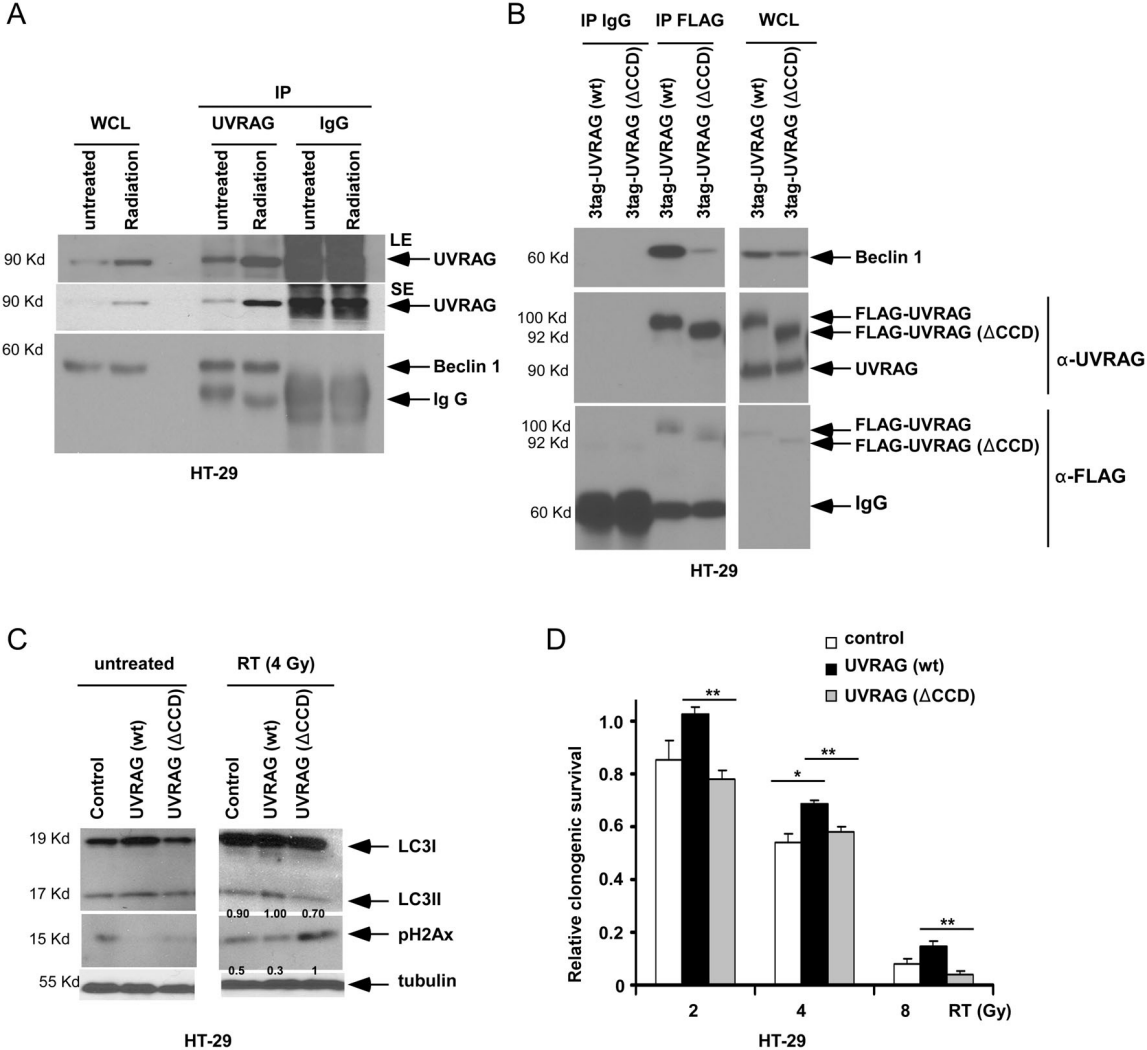
*UVRAG ΔCCD* reduces binding to Beclin 1 and promotes DNA double strand breaks. *A,* Immunoprecipitation of UVRAG followed by probing for Beclin 1 was performed in HT-29 cell lysates (4 h) following radiation (4 Gy) *vs* untreated cells. Normal IgG was utilized as a control for antibody specificity. Both short (SE) and longer (LE) exposures are shown for UVRAG. *B,* HT-29 cells overexpressing *UVRAG* wild-type (wt) or a deletion mutant at its coil-coil domain (ΔCCD), both labeled with a three-tandem-tag [3tag: s-tag, 2XFLAG and streptavidin binding protein (SBP)], were subjected to immunoprecipitation for FLAG. Precipitated proteins were probed using antibodies against Beclin 1, UVRAG or FLAG. Normal IgG was utilized as a control. *C,* Cell lysates from irradiated (4 Gy) cells were probed for LC3I-II and γH2Ax at 24 h post-irradiation by immunoblotting. Stable *UVRAG* wt or *ΔCCD* mutant cells were utilized here and in Fig. 5B. *D*, Cells with wt *UVRAG* or the *UVRAG ΔCCD* mutant vs empty vector control were treated with vehicle or radiation, and long-term clonogenic survival was determined. The data were normalized relative to untreated cells for each cell phenotype. Data are presented as mean ± standard deviation for experiments performed in triplicate. Statistical significance was determined by a two-sided Student’s t test and defined as *P<0.05.

Recent evidence indicates that decreased expression of *UVRAG* seen in some cancers may render tumor cells vulnerable to chromosomal damage [34]. We found that *Beclin 1* siRNA can potently reduce UVRAG expression in the presence or absence of radiation (Fig. 4A). Knockdown of *UVRAG* by siRNA increased radiation-induced DSBs (γH2Ax) (Fig. 4B), levels of pATM (Fig. 4C), and caspase-3 cleavage compared to control siRNA (Fig. 4B). To determine whether *Beclin 1* has an additive effect with *UVRAG* on regulation of radiation-induced DNA damage and apoptosis, we compared cells with knockdown of *UVRAG vs* those with double knockdown of *Beclin 1* and *UVRAG*. While similar levels of γH2Ax and pCHK2 were found, irradiated cells with double-knockdown showed increased caspase-3 cleavage suggesting that modulation of apoptosis by Beclin 1 occurs independently of *UVRAG* (Fig. 4D). We then studied the interaction between Beclin 1 and UVRAG in control and irradiated cell lines. Immunoprecipitated UVRAG was shown to associate with Beclin 1 in the presence or absence of radiation (Fig. 5A).

In HT-29 cells where UVRAG was immunoprecipitated, its induction by radiation was observed compared to untreated cells and UVRAG was also shown to bind to Beclin 1 (Fig. 5A). To study the impact of the Beclin 1 and UVRAG interaction in the regulation of radiosensitivity, we generated HT-29 cells that stably express wild-type (wt) *UVRAG* or *ΔCCD* mutants that mediates its interaction with Beclin 1 [25]. The *UVRAG ΔCCD* mutant cells showed near complete loss of binding to Beclin 1 in contrast to UVRAG wt cells (Fig. 5B). Ectopic wt *UVRAG* was shown to enhance LCI-II conversion in irradiated cells (Fig. 5C). We then sought to determine whether the *UVRAG ΔCCD* can cause loss/attenuation of autophagy induction by radiation. *UVRAG ΔCCD* cells showed a modest reduction in radiation-induced LC3I-II conversion compared to wt cells (Fig. 5C) that may be related to the co-existence of endogenous UVRAG. Cells with the UVRAG *ΔCCD* were more susceptible to radiation-induced DSBs, indicated by increased γH2Ax (Fig. 5C) compared to wt *UVRAG* and empty vector control cells. Furthermore, cells with the *UVRAG ΔCCD* were more susceptible to radiation-induced cell death shown in a long-term clonogenic survival assay compared to UVRAG wt cells (Fig. 5D).

### *Beclin 1* and *UVRAG* Regulate Centrosome Stability

**Figure 6:**
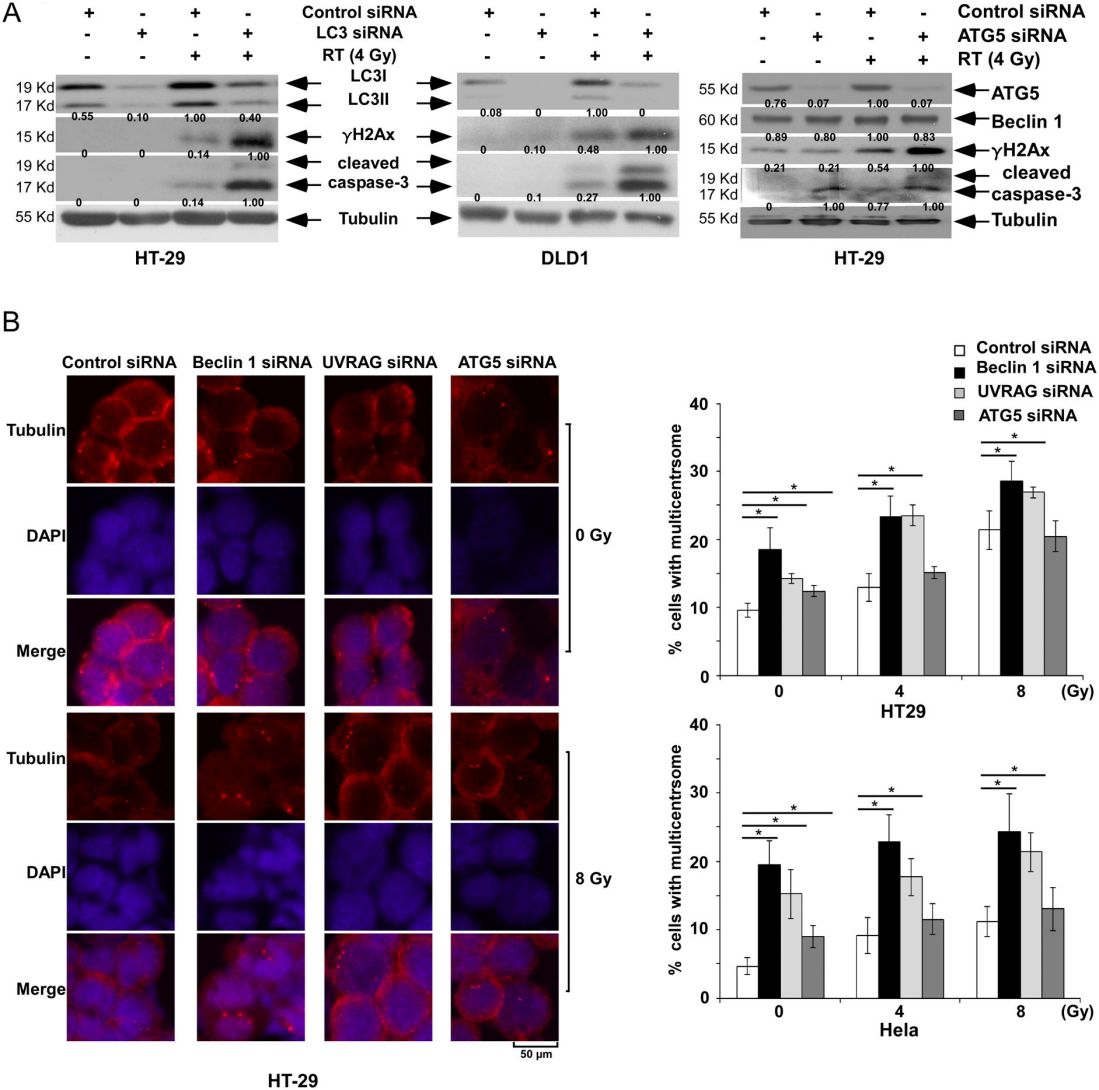
*Beclin 1* or *UVRAG* suppression induces centrosome amplification. *A,* Effect of knockdown of *LC3* (*left*) or *ATG5* (*right*) on markers of DSBs (γH2Ax), apoptosis (caspase-3), and autophagy (LC3I-II conversion) in HT-29 and/or DLD1 cells exposed to radiation *vs* control 24 h post-irradiation. *B*, Centrosome number was determined by immunofluorescence in *Beclin 1*, *UVRAG,* or *ATG5* knockdown cells (HT-29 or HeLa) treated with radiation (4, 8 Gy) *vs* control. Cells were then stained for γ-tubulin (red color) and representative images are shown (*left*). The percentage of cells with more than two centrosomes (multi-centrosomes) was counted and plotted (*right*). Data are presented as mean ± standard deviation for experiments performed in triplicate. Statistical significance was determined by a two-sided Student’s t test and defined as *P<0.05 as compared to the control cells.

Although cells with defective autophagy are prone to genomic instability, evidence supporting a role for autophagy in genome protection is limited and the role of Beclin 1 or UVRAG, if any, is poorly understood. We examined the ability of autophagy regulators to mediate genome protection by analysis of centrosome amplification. Centrosome amplification has been detected in human cancer cells with DNA damage induced by ionizing radiation or cytostatic drugs [35], and can lead to mitotic failure and subsequent cell death [36]. Autophagy inhibition by suppression of *ATG5* or *LC3* by siRNA were shown to enhance radiation-induced γH2Ax and caspase-3 cleavage (Fig. 6A), indicating the ability of autophagy to regulate these processes. We then determined whether Beclin 1 and/or UVRAG can regulate centrosome stability. In untreated HT-29 or Hela cells, we found a statistically significant increase in centrosome number following knockdown of *Beclin 1* or *UVRAG* and to a lesser extent for ATG5, compared to control siRNA shown by γ–tubulin immunofluorescence (Fig. 6B). In irradiated cells, a statistically significant increase in centrosome number was limited to cells with knockdown of *Beclin 1* or *UVRAG,* but not *ATG5* (Fig. 6B). These findings suggest that Beclin 1 and UVRAG may regulate centrosome stability independently of autophagy.

## Discussion

While the role of Beclin 1 and UVRAG in DNA damage has been studied in the setting of tumorigenesis [4], [13], [19], little is known about the molecular details of their role in tumor cell response to cancer therapy. Understanding cellular mechanisms of resistance to DNA damage and repair responses are critical to improving therapeutic outcomes in cancer patients. Appropriate execution of DNA DSB repair is critical for tumor cell survival following DNA damage and for maintenance of genomic stability. We found that Beclin 1 and its cofactor UVRAG can regulate the DNA damage/repair response and centrosome stability in human CRC cells. Specifically, suppression of *Beclin 1* or *UVRAG* was associated with the accumulation of DSBs and with increased apoptotic cell death in response to radiation ± 5-FU, indicating their ability to regulate these processes. A mechanism by which *UVRAG* and *Beclin 1* regulate the DNA damage response is suggested by the finding that suppression of either gene significantly increased the number of irradiated cells with nuclear foci expressing 53BP1 which contributes to NHEJ by interacting with chromatin at DSB sites to regulate 5′ end resection [31]. This observation is consistent with data in *53BP1*-deficient mice that display hypersensitivity to irradiation and exhibit chromosomal abnormalities indicative of DNA repair defects [37]. In contrast to 53BP1, we found that the number of RAD51 nuclear foci was unaffected by *Beclin 1* or *UVRAG* suppression, although further study is needed to determine the preferential involvement of NHEJ *vs* HR in this setting. Since Beclin 1 stability is controlled by ubiquitination, inhibiting deubiquitinases that downregulate Beclin 1 has been shown to disable the cytoprotective effect of Beclin 1. Similar to *Beclin 1* knockdown, we found that suppression of the ubiquitin-specific peptidase, USP10, or a small molecule inhibitor of the deubiquitinases USP10 and USP13, i.e., spautin-1 [33], can increase radiation-induced DSBs and promote tumor cell death. Recent data demonstrate a close relationship between Beclin 1 and p53 via the deubiquitinases USP10 and USP13 [33]. Since USP10 mediates the deubiquitination of p53, regulation of the deubiquitinase activity of USP10 and USP13 by Beclin 1 may provide a mechanism by which it can control the p53 protein levels. However, the relevance of these findings to cells with mutant *p53*, as used in this study, is unknown. While we utilized knockdown approaches and an autophagy inhibitor (spautin), we acknowledge that certain autophagy regulators can directly or indirectly regulate cellular processes that are independent of autophagy [13], [38].

We found that suppression of *Beclin 1* was associated with downregulation of UVRAG, consistent with evidence that the stability of components of the PI(3)KC3 complex that regulates autophagy are inter-dependent at a post-transcriptional level [3], [4]. To determine the functional overlap among these genes, we generated cells with dual knockdown of *UVRAG* and *Beclin 1* which was shown to increase apoptosis, but not DSBs, compared to *UVRAG* knockdown alone. This finding suggests that Beclin 1 can regulate apoptosis independently of UVRAG. Relevant to this observation, Beclin 1 contains a BH3 domain that can be bound to and inhibited by Bcl-2 or Bcl-x_L_ proteins that can inhibit autophagy and apoptosis [2]. We found that the ability of UVRAG to regulate the DNA damage response depends upon its interaction with Beclin 1. Our *UVRAG ΔCCD* mutant showed markedly impaired binding to Beclin resulted in a greater extent of radiation-induced DSBs compared to wild type *UVRAG* and empty vector cells. Clonogenic survival in irradiated cells was significantly reduced in *ΔCCD* compared to *UVRAG* wild type but not empty vector cells, suggesting that other factors or residual binding to Beclin 1 may be responsible. It was recently shown that UVRAG binding to DNA-PK is independent of Beclin 1 and that DNA-PK localizes to sites of NHEJ, suggesting that it maintains genomic stability independently of its binding to Beclin 1 [13]. Furthermore, the carboxy terminus of *UVRAG* and not CCD was shown to be responsible for this function. The *UVRAG ΔCCD* mutant utilized in our study contains an intact DNA-PK binding domain [13]. While our data suggest that the interaction between Beclin 1 and UVRAG can protect cells from DNA damage, the specific contribution of autophagy to this process should ideally be supported by further evidence in autophagy-deficient cells.

A key observation was that Beclin 1 and UVRAG can regulate centrosome stability in colon cancer cells. Suppression of endogenous *Beclin 1* or *UVRAG* resulted in centrosome amplification, as indicated by an increase the number of cells with multi-centrosomes in both untreated and in irradiated cells. This centrosome amplification has consequences for spindle malformation and chromosome segregation errors [13], [39]. In contrast, knockdown of *ATG5* was not associated with significant increase in centrosome number in the presence or absence of radiation. However, the inability of *ATG5* to regulate centrosome number occurred despite the ability of *ATG5*, *Beclin 1*, and *UVRAG* to regulate DSB repair and apoptosis. This finding suggests that maintenance of centrosome stability by *Beclin1/UVRAG* may be independent of their regulatory role in autophagy which was recently shown for *UVRAG* [13]. UVRAG was shown to directly interact with the centrosome protein CEP63, and *UVRAG* mutants lacking CEP63 binding maintained efficient Beclin 1 interaction. Our finding that Beclin 1 can regulate centrosome stability is supported by its nuclear localization in that it contains a leucine-rich nuclear export signal motif [40]. Together, our data demonstrate a role for Beclin 1 and UVRAG in the maintenance of genomic stability through mechanisms involving DSB repair and centrosome stability. Modulation of centrosome amplification has therapeutic implications in that increasing centrosome number by disabling *Beclin 1* or *UVRAG* can increase susceptibility to radiation-induced cell death [36].

In summary, we found that *Beclin 1* and *UVRAG* regulate the DNA damage/repair response that may utilize NHEJ to repair DSBs in irradiated colorectal cancer cells. The ability of UVRAG to regulate DNA damage/repair was dependent upon its Beclin 1 interaction domain since disruption of binding comprised their ability to protect against DSBs. The ability of *Beclin 1* and *UVRAG* to regulate DNA damage/repair and to regulate centrosome number indicates that these genes play a role in maintenance of genomic stability.
